# Mr. Ring's Case of Angina Pectoris

**Published:** 1807-01

**Authors:** John Ring

**Affiliations:** New Street, Hanover Square


### *To the Editors of the Medical and Physical Journal.*

GENTLEMEN,

THE following case of Angina Pectoris, which lately occurred in my practice, is a confirmation of Dr. Jenner's opinion, that an ossification of the coronary arteries is a frequent cause of the disorder.

About the middle of June last, the subject of it, the late Mr. Witherington, of Old Burlington Street, aged sixty-seven, was attacked, while walking, with a shortness of breath, and a tightness across his chest. I was soon after applied to; and he informed me, that he had been in some degree indisposed ever since the month of February; when he had caught a violent cold, in consequence of a long attendance as a juryman, at Westminster ster Hall. He had always lived in a very temperate manner, and enjoyed a good state of health; and had been a great walker.

Symptoms of an inflammatory diathesis appearing, the antiphlogistic plan of treatment was pursued; by which his complaints were greatly relieved. He then went into the country; and, on his return, called on me to inform me that he was well, except a slight degree of dyspnœa.

In the first week of September, I called on him; and was much concerned to find him labouring under decided symptoms of Angina Pectoris. Whenever he walked a moderate distance, he was suddenly seized with a painful constriction of the chest; which also extended itself to the insides of the fore-arms. I advised him to have the opinion of some eminent physician; in consequence of which Dr. Reynolds was consulted; but as he left town soon afterwards, Dr. Bradley was applied to; who attended him with me till his death.

Both these gentlemen agreed with me in opinion, concerning the nature of the case; but it was one in which nothing could be done to remedy, and but little to alleviate, the sufferings of the patient:

——————— cessere magistri
Phillyrides Chiron, Amythaoniusque Melampus.

Apprehending that his dissolution would be sudden, and that it would happen at no great distance of time, I apprised Mrs. Witherington of the circumstance; and my prediction was soon verified. On the 2d of October, while standing still, he was attacked with a syncope; and again, in a slighter degree, the next morning. Nevertheless, in the forenoon he conversed with Dr. Bradley; and seemed cheerful, and composed. He ate a hearty dinner; and when some of the family, who had left the room a few minutes, returned, they thought he was sleeping in his chair; but on trying to wake him, they found he was dead.

Two days afterwards, leave having been obtained, the body was opened by Mr. Brodie, in the presence of Dr. Bradley and myself, and the appearances were as follows.

The lungs were perfectly free from disease. The pericardium contained no more fluid than usual. The heart, when removed with its vessels, was remarkably loose and flabby in its texture. The muscular parietes were much thinner than natural; and the cavities considerably dilated.

The tricuspid valves, and the semilunar valves of the pulpulmonary artery, were in a natural state. The mitral valves, and the semilunar valves of the aorta, were in different parts diseased and thickened; as if in an incipient state of ossification.

The left coronary artery was ossified to a considerable extent; forming a complete bony tube, two inches in length. The right coronary artery was ossified at its origin; but the disease did not extend above half an inch.

I cannot conclude this article without recommending to every one who wishes to understand the nature of this disorder, a careful perusal of the remarks on the subject, published by the late Dr. Heberden in the Medical Transactions; and of Dr. Parry's learned and elaborate Treatise, entitled, "An Inquiry into the Symptoms and Causes of the Syncope Anginosa, commonly called Angina Pectoris." A ready perception of such a disease is peculiarly desirable; as it will tend to preserve the friends of the patient from the violence of the shock, which a sudden and unexpected death would occasion; and to secure the medical practitioner from censure.

---

I have lately received from Dr. Jenner, a farther account of the cases of cow-pox that occurred last spring, at a farm near Berkeley, of which some particulars have already appeared in this Journal; and in which the disorder was clearly traced to the grease. Mr. Cole, the occupier of the farm, having five years before had a horse in his stable labouring under this disorder, was cautioned by a veterinary practitioner, not to suffer the man who dressed him to milk his cows; but no regard was paid to this advice, and he continued to milk as usual.

Dr. Jenner, happening to be at Berkeley at the time, and Mr. Cole living within a mile of him, he was a witness to the confusion into which the farm was thrown by this inadvertency; for scarcely a cow or a servant in the farm escaped infection.

In the month of April last, Mr. Cole happened to have a horse in the same complaint, and as the consequences of the former want of precaution were still fresh in his memory, he strictly charged the boy who dressed the horse, not to milk any of the cows, till he received farther orders. This injunction, however, was not obeyed; for, at the instigation of a fellow servant, he milked three cows, which happened to be in a meadow by themselves, for several days days; and only these. They were all presently infected, together with the boy. Dr. Jenner carefully watched its progress; and never saw the disorder in a more regular form.

Hearing that Dr. Adams, of the Small-pox Hospital, was in the neighbourhood, he invited him to see these cases; and to hear the history of them from the mouths of the farmer and his servants; at which he expressed the utmost satisfaction. He was also furnished with virus from one of the pustules; with which, as he has since informed Dr. Jenner, he produced the genuine cow-pock, on his return to London.

This is the only instance of the cow-pox, which Doctor Jenner heard of in the neighbourhood of Berkeley, in the course of the last spring. The disease is become far more uncommon than formerly; owing to the precautions now observed by the farmers.

He concludes his letter as follows: "If you recollect, in my first treatise on the Variolæ Vaccinæ, I mentioned an instance where the disease sprung up at a farm from the fluid formed in a cluster of erysipelatous or herpetic vesicles, on the thigh of a colt, at Mr. Millet's, at Rockhampton. An appearance in some degree similar to this, I observe, is excited in the integuments surrounding the cracks or fissures in the heels of horses; and from this vesiculated surface, I conceive, and not from the cracks themselves, oozes the peculiar fluid. This, if made public, may prove a useful hint to those, who may be inclined to make farther experiments on the subject.

---

The next article of intelligence which I beg leave to communicate, is contained in the following Supplement to the Madrid Gazette, dated October 14, 1806.

"On Sunday, the 7th of September last, Dr. Francis Xavier Balmis, Surgeon Extraordinary to the King, had the honour of kissing his Majesty's hand, on occasion of his return from a voyage round the world, executed with the sole object of carrying to all the possessions of the Crown of Spain, situated beyond the seas, and to those of several other nations, the inestimable gift of Vaccine Inoculation, His Majesty has enquired, with the liveliest interest, into all that materially related to the expedition; and learned, with the utmost satisfaction, that its result has has exceeded the most sanguine expectations which were entertained at the time of the enterprize.

This undertaking had been committed to the diligence of several Members of the Faculty, and subordinate persons; carrying with them twenty-two children, who had never undergone the small-pox; selected for the preservation of the precious fluid, by transmitting it successively from one to another, during the course of the voyage. The expedition set sail from Corunna, under the direction of Balmis, on the 30th of November, 1803. It made the first stoppage at the Canary Islands, the second at Porto-Rico, and the third at the Caracas. On leaving that province, by the port of La Guayra, it was divided into two branches: one part sailing to South America, under the charge of the Subdirector Don Francis Salvani; the other, with the Director Balmis on board, steering for the Havannah and thence for Yucatan. There a subdivision took place: the professor Francis Pastor proceeding from the port of Sisal, to that of Villahermosa, in the province of Tobasco, for the purpose of propagating Vaccination in the district of Ciudad Real de Chiapa, and on to Goatemala, making a circuit of four hundred leagues, through a long and rough road, comprising Oaxaca; while the rest of the expedition, which arrived without accident at Vera-cruz, traversed not only the Vice-royalty of New Spain, but also the interior provinces; whence it was to return to Mexico, which was the point of re-union.

" This precious preservative against the ravages of the small-pox has already been extended through the whole of North America, to the coasts of Sonora and Sinaloa, and even to the pagans and new converts of Pimeria Alta. In each capital a Council has been instituted, composed of the principal Authorities, and the most zealous Members of the Faculty; charged with the preservation of this invaluable specific, as a sacred deposit, for which they are accountable to the King, and to posterity.

" This being accomplished, it was the next care of the Director to carry this part of the expedition from America to Asia, crowned with the most brilliant success, and, with it, the comfort of Humanity. Some difficulties having been surmounted, he embarked in the port of Acapulco for the Philippine Islands; that being the point at which, if attainable, it was originally intended that the undertaking should be terminated.

" The bounty of Divine Providence having vouchsafed to second the great and pious designs of the King, Balmis happily happily performed the voyage in little more than two months; carrying with him, from New Spain, twenty-six children, destined to be vaccinated in succession, as before; and as many of them were infants, they were committed to the care of the Matron of the Foundling Hospital at La Corunna; who, in this, as well as the former voyages, conducted herself in a manner to merit approbation. The expedition having arrived at the Philippines, and propagated the specific in the islands subject to His Catholic Majesty; Balmis, having concluded his philanthropic commission, concerted with the Captain General the means of extending the beneficence of the king, and the glory of his august name, to the remotest confines of Asia.

"In point of fact, the cow-pox has been disseminated through the vast Archipelago of the Visayan Islands; whose Chiefs, accustomed to wage perpetual war with us, have laid down their arms, admiring the generosity of an enemy, who conferred upon them the blessings of health and life, at the time when they were labouring under the ravages of an epidemic small-pox. The principal persons of the Portuguese colonies, and of the Chinese empire, manifested themselves no less beholden, when Balmis reached Macao and Canton; in both which places he accomplished the introduction of fresh virus, in all its activity, by the means already related; a result, which the English, on repeated trials, had failed to procure, in the various occasions when they brought out portions of matter in the ships of their East India Company; which lost their efficacy on the passage, and arrived inert.

"After having propagated the Vaccine Inoculation at Canton, as far as possibility and the political circumstances of the empire would permit, and having confided the further dissemination of it to the Physicians of the English factory at the above-mentioned port, Balmis returned to Macao, and embarked in a Portuguese vessel for Lisbon; where he arrived on the 15th of August. In the way he stopped at St. Helena, in which, as in other places, by dint of exhortation and perseverance, he prevailed upon the English to adopt the astonishing antidote, which they had undervalued for the space of more than eight years, though it was a discovery of their nation, and though it was sent to them by Jenner himself.

"Of that branch of the expedition which was destined for Peru, it is ascertained that it was shipwrecked in one of the mouths of the River de la Magdalena; but having derived immediate succour from the natives, and from the Magistrates adjacent, and from the Governor of Carthagena, the Subdirector, the three Members of the Faculty who accompanied him, and the children, were saved, with the fluid in good preservation, which they extended in that port, and its province, with activity and success. Thence it was carried to the isthmus of Panama, and persons, properly provided with all necessaries, undertook the long and painful navigation of the River de la Magdalena; separating, when they reached the interior, to discharge their commissions in the towns of Teneriffe, Mompox, Ocana, Socorro, San Gil y Medelin, in the valley of Cucuta, and in the cities of Pamplona, Giron, Tunja, Velez, and other places in the neighbourhood, until they met at Santa Fe: leaving every where suitable instructions for the Members of the Faculty, and, in the more considerable towns, regulations conformable to those rules which the Director had prescribed for the preservation of the virus; which the Viceroy affirms to have been communicated to *fifty thousand* persons, without one unfavourable result. Towards the end of March, 1805, they prepared to continue their journey in separate tracks, for the purpose of extending themselves, with greater facility and promptitude, over the remaining districts of the Viceroyalty, situated in the road of Popayan, Cuenca, and Quito, as far as Lima. In August following they reached Guayaquil.

"The result of this expedition has been, not merely to propagate vaccination amongst all people, whether friends or enemies, among Moors, Visayans, and Chinese, but also to secure to posterity, in the dominions of his Majesty, the perpetuity of so great a benefit; partly by means of the Central Committees that have been established, and, partly, by the discovery of indigenous matter in the cows of the valley of Atlixco, near the city of Puebla de los Angeles, by Balmis, in the neighbourhood of that of Valladolid de Mechoacan, by the Adjutant Antonio Gutierrez, and in the district of Calabozo, in the province of Caracas, by Don Carlos de Pozo, the Physician of the residence.

"A multitude of observations, which will be published without delay, respecting the developement of the cowpock in various climes, and its efficacy, not merely in preventing the Natural Small-pox, but in curing, at the same same time, other morbid affections of the human frame, will manifest how important the consequences of an expedition, which has no parallel in history, will prove to the cause of humanity.

"Though the object of this undertaking was limited to the communication of the cow-pock in every quarter, the instruction of Practitioners, and the establishment of regulations, which might serve to render it perpetual,—nevertheless, the Director has omitted no means of rendering his services beneficial, at the same time, to Agriculture and the Sciences. He brings with him a considerable collection of exotic plants. He has caused drawings to be made of the most valuable subjects in Natural History. He has amassed much important information; and, among other claims to the gratitude of his country, not the least consists in having imported a valuable assemblage of trees and vegetables, in a state to admit of propagation; and which, being cultivated in those parts of the peninsula that are most congenial with their growth, will render this expedition as memorable in the annals of Agriculture, as in those of Medicine and Humanity. It is hoped that the Subdirector and his coadjutors, appointed to carry these blessings to Peru, will shortly return by way of Buenos-Ayres; after accomplishing their journey through that Vice-royalty, the Vice-royalty of Lima, and the districts of Chili and Charcas; and that they will bring with them such collections and observations as they have been able to acquire, according to the instructions given by the Director; without losing sight of the philanthropic commission which they received from his Majesty, in the plenitude of his zeal for the welfare of the human race."

This intelligence, pleasing and satisfactory as it is, must excite humiliating reflections in the breast of every Briton; and I cannot help joining in sentiment with my respectable correspondent from whom I received it. He says, "Would to Heaven, our own country could have boasted of fitting out a philanthropic expedition like this!"

I am, &c.

JOHN RING.

*New Street, Hanover Square.*

*Observations*

